# Computed tomography and magnetic resonance imaging of hydatid disease: A pictorial review of uncommon imaging presentations

**DOI:** 10.1016/j.heliyon.2021.e07086

**Published:** 2021-05-22

**Authors:** Bita Abbasi, Reza Akhavan, Afshar Ghamari Khameneh, Gisoo Darban Hosseini Amirkhiz, Hossein Rezaei-Dalouei, Shamim Tayebi, Jahanbakhsh Hashemi, Behzad Aminizadeh, Sanam Darban Hosseini Amirkhiz

**Affiliations:** a Department of Radiology, Faculty of Medicine, Mashhad University of Medical Sciences, Mashhad, Iran; b Department of Emergency Medicine, Faculty of Medicine, Mashhad University of Medical Sciences, Mashhad, Iran; c Research Center for Prevention of Cardiovascular Disease, Institute of Endocrinology and Metabolism, Iran University of Medical Sciences (IUMS), Tehran, Iran

**Keywords:** Hydatid cyst, Computed tomography, Magnetic resonance imaging

## Abstract

Hydatid disease (HD), also known as echinococcal disease or echinococcosis, is a worldwide zoonosis with a wide geographic distribution. It can be found in almost all parts of the body and usually remains silent for a long period of time. Clinical history can be varied based on the location, size, host immune response, and complications.

The most common imaging modalities used for diagnosis and further evaluations of HD are ultrasound, computed tomography (CT), and magnetic resonance imaging (MRI). Although conventional radiography may be the first used tool, rarely can lead to a definite judgment. Clinical indications and cyst location may alter the choice of imaging. MRI and CT would be useful when the involved area is inaccessible for ultrasound or surgical treatment is required. CT is particularly valuable for osseous organ involvements and the presence of calcifications in the cyst and also demonstrates the size, number, and local complications. MRI can differentiate HD from neoplasms in cases with an unusual appearance on imaging. Moreover, it is preferable in biliary or neural involvements. Besides, more detailed images of MRI and CT could help to resolve the diagnostic uncertainty.

Imaging is the main stem for HD diagnosis. Brain, orbit, muscle, bone, and vascular structures are less commonly involved areas. Familiarity with typical clinical presentation, CT scan and MR imaging findings of HD in this sites facilitate the radiologic diagnosis and guiding appropriate treatment.

## 1 Introduction

Hydatid disease (HD), also known as echinococcal disease or echinococcosis, is a worldwide zoonosis with a wide geographic distribution that is seen in almost all countries [1, 2, 3]. The disease is highly endemic in some parts of North and East Africa, Europe, Asia, the Middle East, and South America [4]. Despite its global spread, the World Health Organization (WHO) classifies the disease as a neglected tropical disease with an annual financial burden of over US$ 3 billion worldwide [3]. The total number of involved cases is most likely being underestimated due to its asymptomatic course and lack of clinical suspicion.

Echinococcosis is caused by the larval stage of Echinococcus. Echinococcus granulosus species is responsible for more than 95% of human HD. Alveolar echinococcosis is a less common form of the disease which is caused by E. Multilocularis [5, 6]. In rare cases, Echinococcus vogeli and Echinococcus oligarthrus are responsible for polycystic echinococcosis [7].

Parasite's life cycle involves two hosts. The definitive hosts are usually dogs and less commonly other carnivores. The adult tapeworm lives in the small intestine of the definite host and attaches to the mucosa by hooklets. The eggs are released within the bowel and passed through feces [8]. Sheep are the most common intermediate hosts that ingest the eggs while grazing on plants in contaminated grounds. The eggs lose their protective layer within the duodenum, the embryos get released and pass the intestinal wall to enter the portal blood flow and form hepatic cysts [9]. The life cycle is complete when the definitive host eats the viscera of the intermediate host. Human contamination occurs through the fecal-oral route (by ingesting infected water or vegetables). Human begins act as intermediate hosts [9]. The parasite passes the human intestinal wall and reaches the portal blood flow or lymphatic system. The liver is the first defensive line and so the most commonly involved organ. The cysts grow to about 1 cm within the first six months and then about 3 cm per year according to the host tissue resistance [9].

Hydatid disease can be found in almost all parts of the body. Multiple cysts and multiorgan involvement are seen in 20–40% of patients*.* [10] For inexperienced clinicians, HD could be a tough clinical challenge. It can be asymptomatic for many years due to slow-growing nature of cysts [9]. In general, clinical presentations are nonspecific. Variable symptoms can be seen based on the location, size, host immune response, and complications such as superimposed bacterial infections and cyst rupture. Cyst rupture or fistulization into adjacent organ leading to anaphylactic shock is the most ominous clinical manifestation of the disease [2, 11, 12].

History of animal contact (especially dogs) and living in a sheep-raising or cattle-raising rural areas are generally present. Dairy farming seems to be an important risk factor. Sixty percent of patients are involved while practicing vocational or part-time farming, gardening, forestry, or hunting [13].

Different serological tests have been introduced for HD diagnosis. Although none of them is the definitive method, they provide supplementary information for case detection and follow-up after treatment [14]. As laboratory findings have a low sensitivity in the brain, musculoskeletal, orbital, and other less common sites of involvement, imaging with a typical history of living in endemic areas is the most helpful diagnostic approach [15]. The most common imaging modalities used for diagnosis and further evaluations of HD are ultrasound, computed tomography (CT), and magnetic resonance imaging (MRI). Although conventional radiography may be the first used tool, rarely can lead to a definite judgment. Ultrasound is the choice for screening, follow-up after treatment, and also cyst staging [16]. Besides the lower costs and innocuous nature, ultrasound simply reveals a water attenuation cyst with a well-defined wall encircling floating membrane, hydatid sands, and vesicles [17]. Clinical indications and cyst location may alter the choice of imaging. For example, MRI and CT would be useful when the involved area is inaccessible for ultrasound or surgical treatment is required. CT is particularly valuable for osseous organ involvements and the presence of calcifications in the cyst and also demonstrates the size, number, and local complications. MRI can differentiate HD from neoplasms in cases with an unusual appearance on imaging. Moreover, it is preferable in biliary or neural involvements. Besides, more detailed images of MRI and CT could help to resolve the diagnostic uncertainty [18, 19].

Imaging plays a critical role in the diagnosis and staging of the disease, and it is crucial for physicians working in endemic areas to be familiar with less common imaging presentations of HD, as it may be easily misdiagnosed and lead to unfavorable outcomes. According to the rising waves of immigration from the endemic areas, it is also essential for physicians in all countries to be familiar with different imaging presentations of the disease.

Treatment of HD is usually pricey and complex. Echinococcosis may entail substantial surgery and/or extended drug therapy. Complete surgical removal with and without albendazole administration is the main stem of HD management, especially in cases with complications or impossible percutaneous drainage [44].

Puncture, aspiration, injection, and re-aspiration (PAIR) is a minimally invasive technique used for definitive treatment in the localized abdominal or soft tissue HD. Cyst contents are aspirated using ultrasound or computed tomography (CT) guidance. In the next step, hypertonic saline solution or absolute alcohol are injected as scolicidal agents. At the final step, cyst contents are re-aspirated. Albendazole should be administrated before and after the procedure to reduce the risk of seeding [20].

This pictorial review emphasizes the imaging features of HD on CT and MRI in uncommon locations including the brain, orbit, muscle, bone, and intravascular. It also aims to discuss the most important aspect of clinical findings, diagnosis, and management.

## 2 Brain

Brain hydatid is very rare and accounts for 1–2% of all intracranial masses, even in endemic countries [21]. Brain HD is more common in children [22, 23]. Symptoms are nonspecific including nausea, vomiting, headache, hemiparesis, visual impairment, and sometimes seizure. Papilledema is usually evident on physical examination [2].

Most intracranial hydatid cysts are supratentorial and in the territory of the middle cerebral artery. The parietal lobes are the most common site of involvement. HD is rarely found within the ventricular system or in the posterior fossa [24].

**Figure 1:**
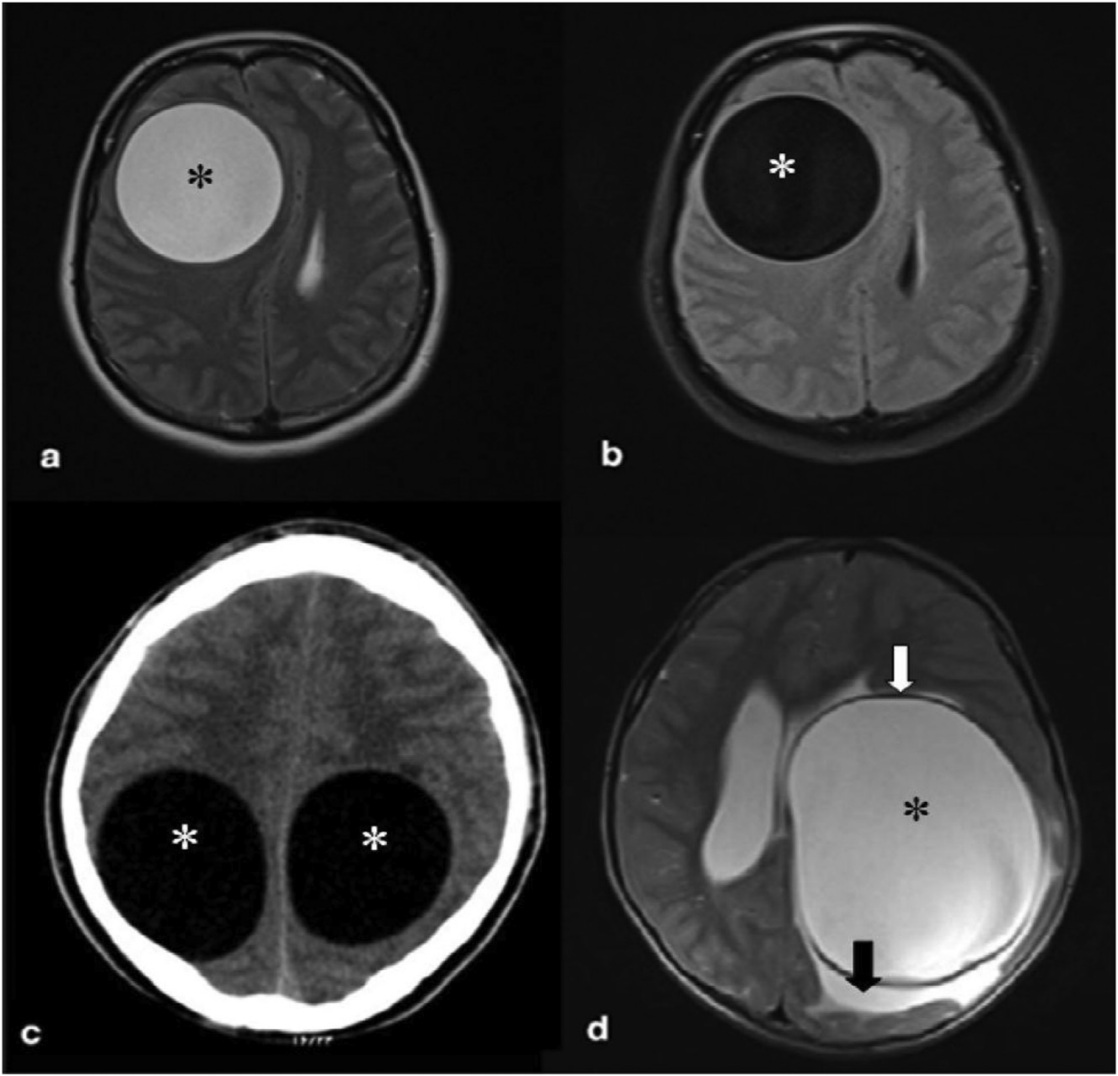
Axial T2-weighted (a) and FLAIR (b) MRI in a young man show a well-defined, round cystic lesion (Asterisk) in the right hemisphere. Axial brain CT scan in a 12-year-old child (c) shows two well-defined cystic lesions (asterisks) in the parietal lobes that were proved to be hydatid cysts after surgical removal. Axial T2-weighted brain MRI (d) in a different patient shows a large intracranial cyst (Asterisk) with low-signal wall (white arrow) and minimal peripheral edema (black arrow). (Photos are from our own patient(s) and they had not been published previously elsewhere.).

**Figure 2:**
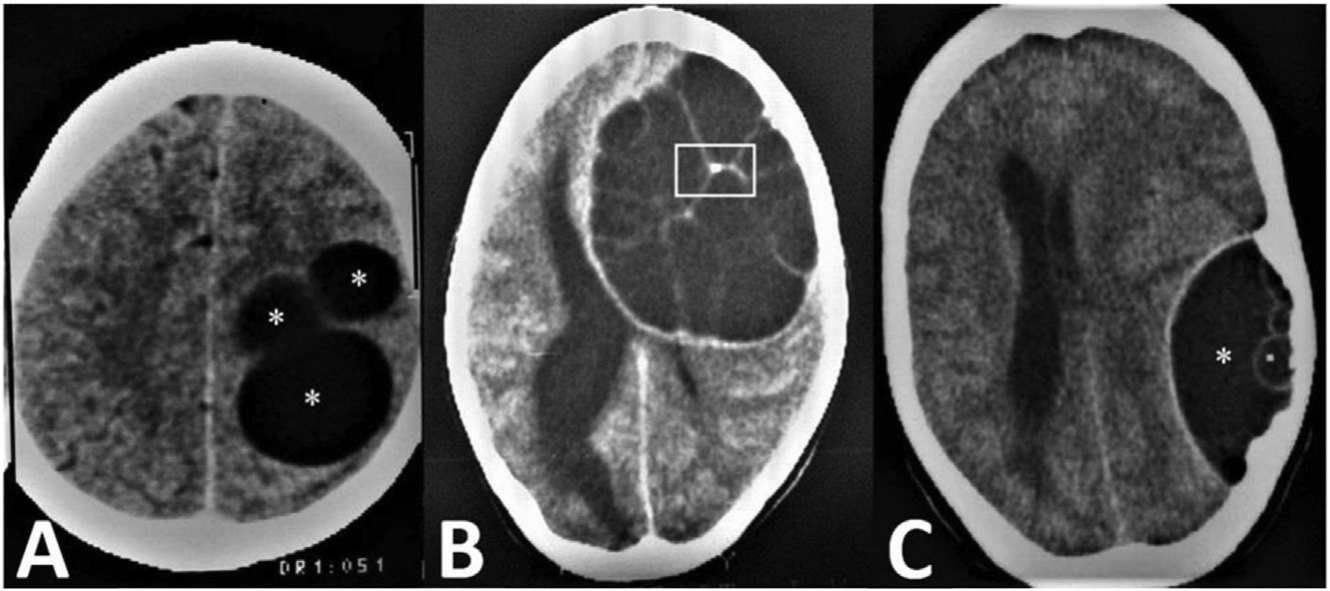
Axial brain CT scans shows A) multiple hydatid cysts (asterisks) in a 45-year-old woman, B) a multiloculated hydatid cyst with septal calcification (rectangle) in a 12-y/o girl, C) post-surgical recurrence of hydatid cyst presenting as a multiloculated extra-axial lesion (Asterisk) in a 40-y/o woman. (Photos are from our own patient(s) and they had not been published previously elsewhere.).

MRI is the imaging modality of choice for brain HD although, CT is also effective. Hydatid cysts in the brain are often unilocular and have cerebrospinal fluid (CSF) signal-intensity and density [9]. Extrinsic compression of the ventricular system results in hydrocephalus. Lack of significant edema around the lesion is a suggestive finding that helps in differentiating brain HD from intracranial abscesses and cystic tumors. The presence of a hypointense rim on the MRI, especially in the T2-weighted sequence, is a characteristic feature of brain hydatid (Figure 1) [25]. On the other hand the cyst border on CT could be isodense or hyperdense compared to the adjacent brain [25]. Less than 1% of cases demonstrate calcifications, which could be resulted from E. Multilocularis infection with cysts containing septations and solid parts [9, 25] or E. Granulosus infection demonstrating septal calcifications (Figure 2C). The multivesicular cysts in the brain is a rare manifestation (Figure 2B, C).

Superimposed bacterial infections or rupture may lead to rim enhancement or perilesional edema [2]. Intracranial hydatid cysts are usually single and can be multiple in cases of spontaneous rupture or trauma (Figure 2A). They usually have no thick capsule in the latter condition. Synchronous involvement of other organs is reported in up to 18% of patients [26].

Arachnoid, porencephalic and epidermoid cysts, pyogenic abscess, the cystic tumor of the brain, and neurocysticercosis are differential diagnoses of brain hydatid cyst. Arachnoid and porencephalic cysts are not round and they are not entirely covered by brain tissue. Other distinguishing features are based on the absence of enhancing rim, perilesional edema, and mural nodules [27].

Surgery is still the mainstay treatment for cerebral HD [28], during which the main purpose is to dislodge the cyst with an intact wall. The Dowling-Orlando technique is the most favored method. In this technique, normal saline infusion between the cyst and surrounding brain is used to dissect the cyst from nearby parenchyma (hydro-dissection) [29]. It is essential to keep craniotomy size large, in order not to injure the cyst wall during the dural entrance, and for careful cortical dissection and identification of cyst border. Hypertonic saline is used in the surgical plane to reduce the risk of relapse. Anthelminthic drugs including Benzimidazoles and Praziquantel have a limited role in cerebral HD treatment and are only used in cases of cyst rupture, systemic involvement, or recurrence [30].

## 3 Orbit

Orbital involvement is seen in less than 1% of hydatid cases [31, 32] but they are the second most common cystic lesion of orbit after dermoid cyst in endemic areas [33]. Orbital HD frequently involves children and young adults [34]. It is usually unilateral and concomitant organ involvement is rare [35].

Proptosis is usually seen and may be painful. Other clinical findings include chemosis, palpebral edema, orbital cellulitis, visual impairment, and restriction of extraocular movements [36]. Slow-growing unilateral and painless proptosis in endemic regions is a highly suspicious indicator of orbital hydatid cyst [35].

**Figure 3:**
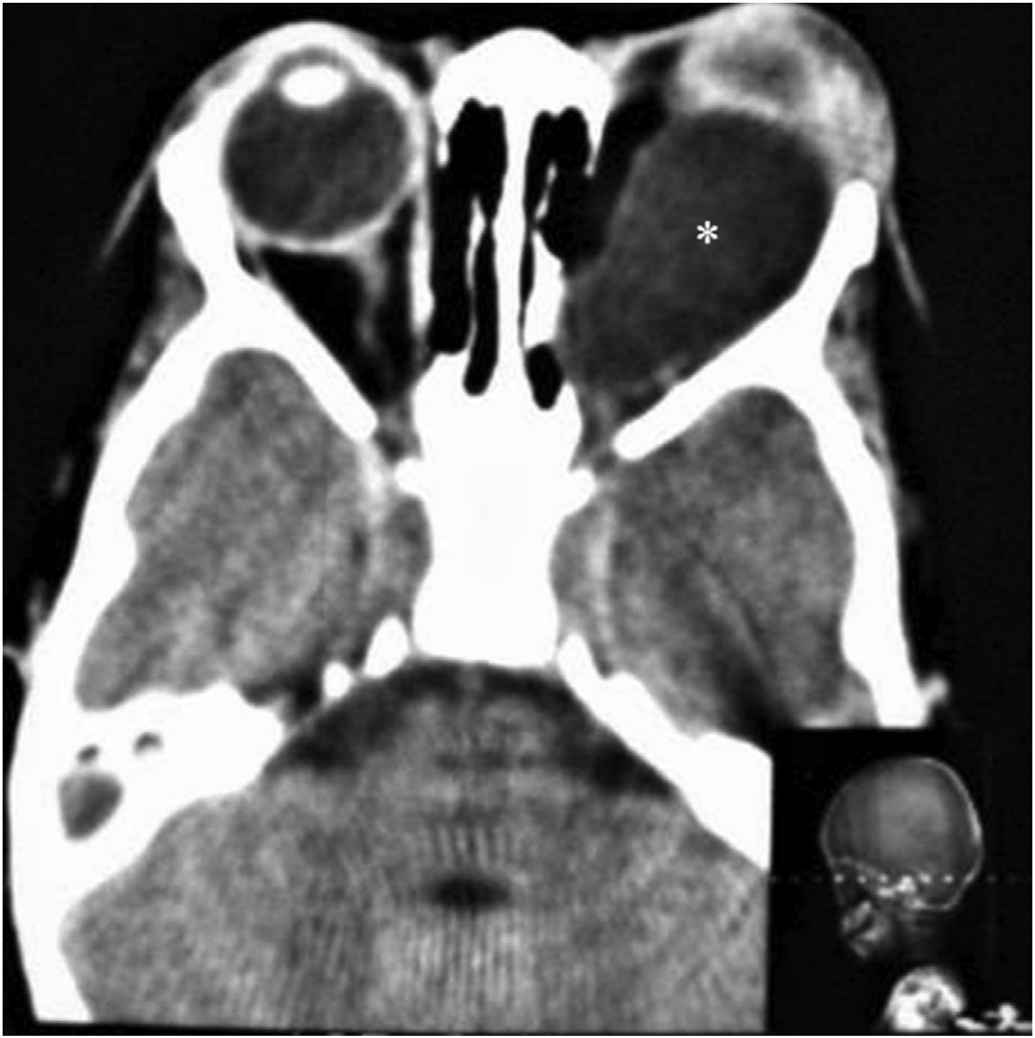
Axial orbital CT scan in a 2-year-old child shows a well-defined hydatid cyst (Asterisk) in the intraconal space of the left orbit. Note the anterior displacement of globe in the involved eye. (Photos are from our own patient(s) and they had not been published previously elsewhere.).

Orbital cysts are typically uniloculated and homogeneous and can be either hypo or hyperdense to vitreous with possible orbital thinning on CT (Figure 3). Superolateral and superomedial angles of the orbit, within or adjacent to the muscular cone are the most common locations [33, 37]. MRI is the most helpful in evaluating the inner structure of the cyst and orbital soft tissue involvement. The cysts are usually hypo- to iso-intense on T1 and hyperintense on T2. After contrast administration, peripheral rim enhancement is typically revealed on both CT scan and MRI [35]. Osseous erosions are best seen on CT [38].

Other cystic pathologies of orbit such as abscess, lacrimal gland cyst or tumor, hematoma, lymphangioma, teratoma, dermoid and epidermoid cysts are the differential diagnosis of orbital HD.

Considering significant sequels of orbital HD in the time of diagnosis, immediate surgery is preferred to avoid irreversible visual loss in most patients, although asymptomatic cases could be managed by pharmaceutical treatment. There is a reported case of cyst eradication by the Albendazole administration. The approach will be changed to cyst disruption, membrane removal, and infusion of solicidal hypertonic saline [34] in cases which complete resection is impossible due to adhesion [39].

## 4 Muscle

Intramuscular hydatid is rare because cyst growth is difficult due to the presence of an unfavorable lactate-containing environment and muscle contractions [40] and occurs in 0.7–0.9% of patients with HD in endemic areas. The tendency towards the muscles of the neck, trunk, and proximal of the extremities is probably due to more vascularity and fewer movements in these muscles [41]. Primary muscle involvement is rare and has been reported in only a few cases [42].

Painless slow-growing palpable mass with or without cellulitis could be a clinical indicator of the HD in extremities [43]. However, compression of the nearby structures, immunologic reactions, and cyst complications can lead to various signs and symptoms [44]. Intramuscular HD could be in the differential diagnosis list for chronic hematoma, abscess, necrotic malignant tumors such as malignant fibrous histiocytoma, and also synovial cyst.

**Figure 4:**
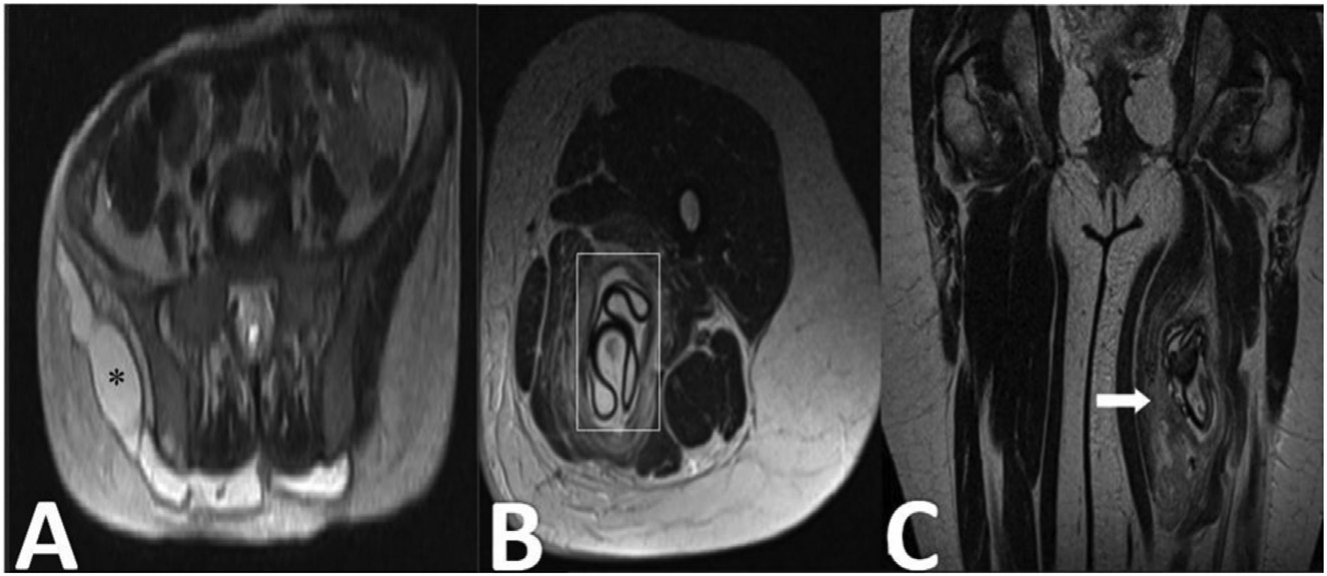
Axial T2 fat-sat MRI (A) of the pelvis shows an extensive cystic lesion in the gluteal soft tissue with low-signal wall (Asterisk). The lesion proved to be hydatid disease. Axial (B) and coronal (C) T2-weighted MRI of the thigh in a middle-aged man shows the typical layered appearance hydatid cyst (rectangle) in a thigh muscle. Note the presence of edema (arrow) adjacent to the hydatid cyst. (Photos are from our own patient(s) and they had not been published previously elsewhere.).

**Figure 5:**
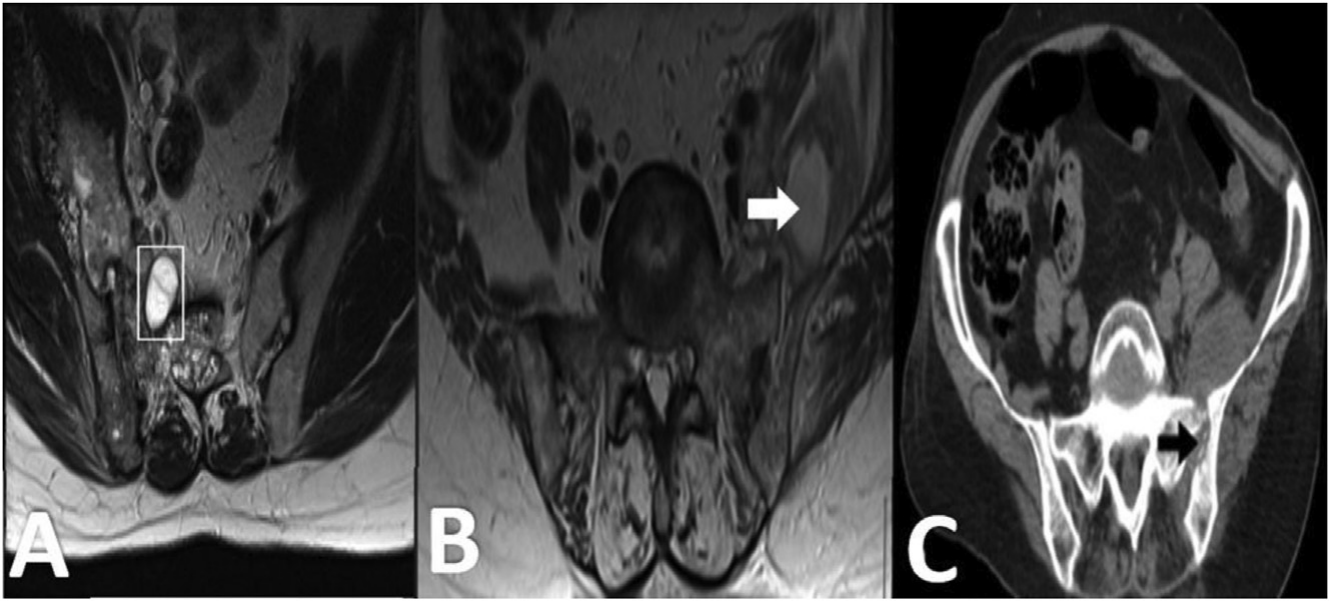
Axial T2W (A) image shows multiloculated lesions in the right iliacus muscle, right iliac wing and sacrum (rectangle). Axial T2-weighted MRI (B) and axial pelvic CT scan (C) show a cystic lesion (white arrow) in the left iliacus muscle with extension into the sacroiliac joint (black arrow). The lesion was biopsied and HD was confirmed histologically. (Photos are from our own patient(s) and they had not been published previously elsewhere.).

Misdiagnosis is common in patients with intramuscular HD as usually serology is negative and imaging, which has the primary role in diagnosis, may show nonspecific findings [45]. Various types of hydatid cysts may be seen in the muscles. Multiple hydatidoses is seen in the cases of previous surgery, trauma, or cyst rupture. Edema and acute inflammation in the soft tissue adjacent to the cyst is probable but uncommon occurrences. Ultrasound is a cost-effective first-line imaging modality for soft tissue masses [46] but most of the time it is not accurate for intramuscular HD diagnosis. Calcification is rare in musculoskeletal HD and CT findings are often atypical so CT is only preferred for evaluation of bone involvements and MRI is the modality of choice [41, 47]. Multivesicular cyst and detached membrane, as well as low signal rim in the T2-weighted sequence, are classic MRI appearance (Figures 4 and 5).

These cysts are commonly treated by surgical removal under localized anesthesia. Neo-adjuvant/adjuvant Albendazole or Mebendazole is administrated for decreasing recurrence [45].

## 5 Bone

**Figure 6:**
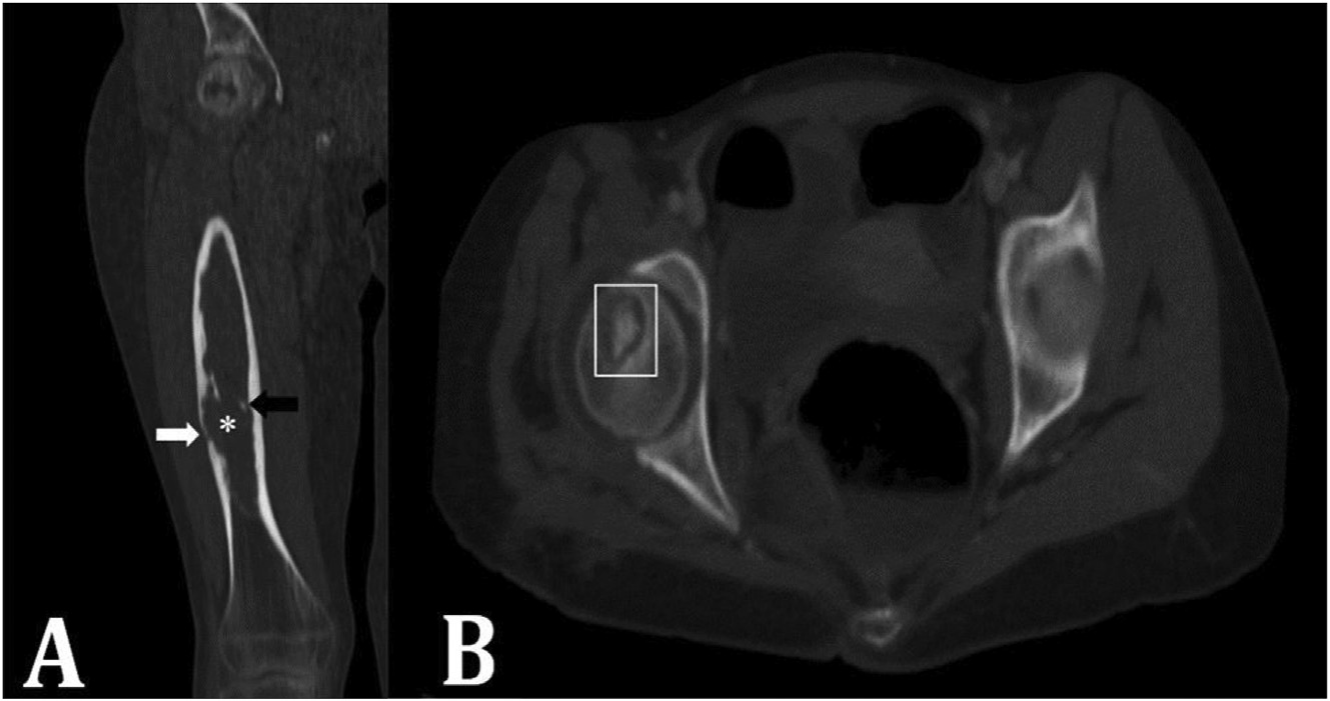
A) Coronal reformatted CT scan of lower limb in a 14-year-old girl with generalized hydatidosis shows expansile intra-osseous lesion (Asterisk) in the right femur. Note the areas of cortical thinning (white arrow) and intralesional calcifications (black arrow). B) Axial CT scan of pelvic bones shows a lytic lesion in the femoral head with internal calcifications (rectangle). Note the intra-articular involvement. (Photos are from our own patient(s) and they had not been published previously elsewhere.).

Bone involvement is rare and accounts for 0.5–2% of HD cases. The most common involved bones are vertebrae (50%), pelvis (21%), femur (16%), tibia (10%), and with lower incidence rates in ribs, skull, scapula, humerus, and fibula [9, 48, 49]. Bone involvement could be primary but lots of chest wall and spinal osseous HDs have resulted from extension of adjacent lung or liver HD [50]. The involved patients can remain asymptomatic for a long period due to the slow growth of the cyst within the bone. The disease is commonly diagnosed in the middle ages and incidence in childhood is rare (Figure 6A) [51].

Pain is the most frequent clinical symptom [52]. Neurological deficits, local swelling, and pathologic fracture are other important clinical findings. It must be emphasized that symptoms have significant overlap with bone malignancy [51, 53]. HD should be considered as a possible cause of spinal cord compression syndrome in endemic areas. Back pain, radiculopathy, paraparesis, paraplegia, local tenderness, sensory disturbance, and sphincter involvement are described in the previously reported cases [54]. The thoracic spinal cord is the most common site (50%) of vertebral involvement, followed by lumbar (20%), sacral (20%), and cervical (10%) areas. Most cases of spinal HD are multiple with direct extension to the adjacent bones [52, 55].

**Figure 7:**
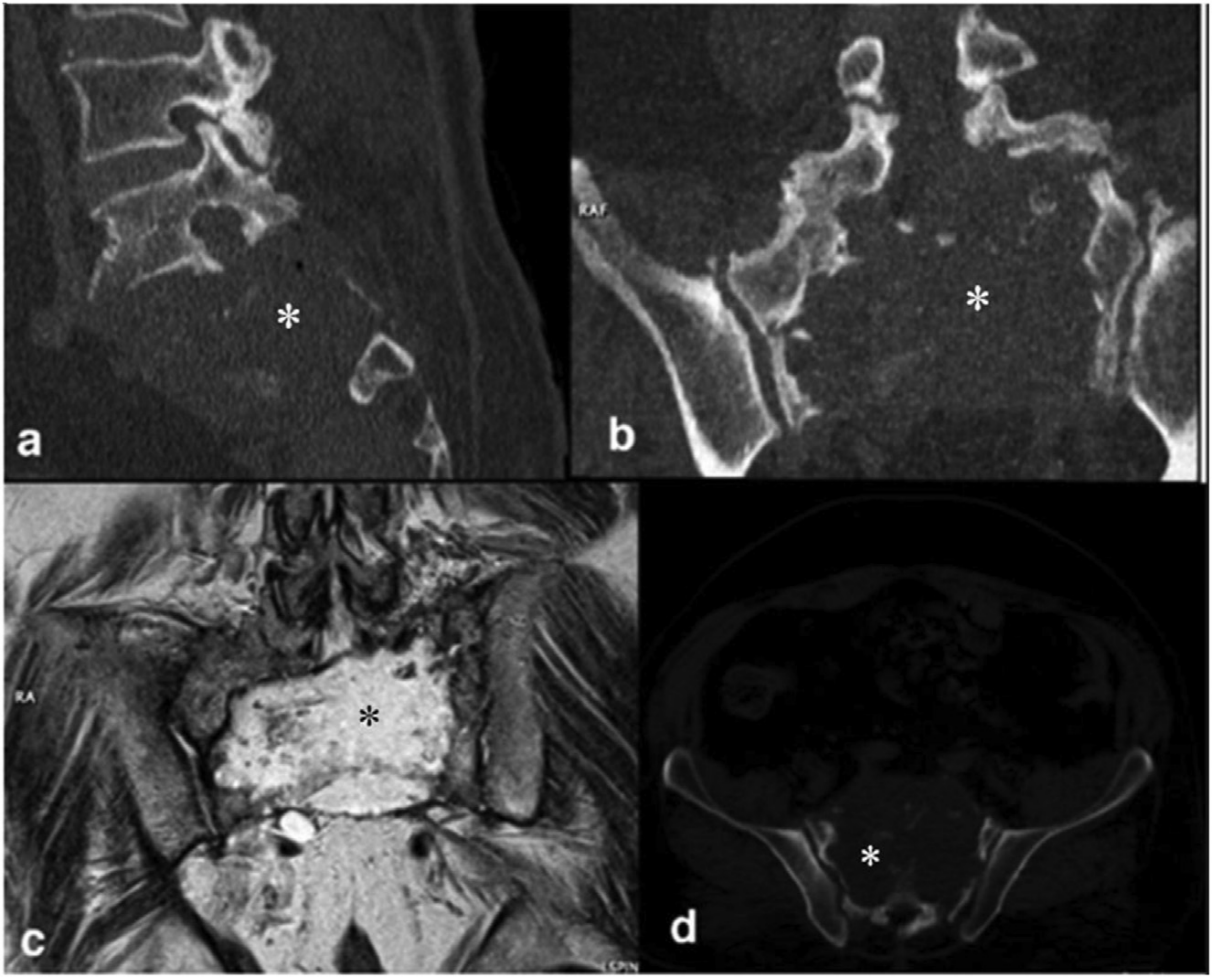
Sagittal multiplanar reconstruction CT scan (a), coronal CT scan (b), coronal T2-weighted MRI (c) and coronal reconstructed CT scan in bone window (d) show a cystic multiloculated hydatid disease in the sacrum (Asterisk). (Photos are from our own patient(s) and they had not been published previously elsewhere.).

HD in the bone lacks pericyst, thus a greater number of patients may represent a positive serologic test result. However, CT and MRI are still the first and best modalities suggesting the diagnosis of osseous HD [52]. The cysts are usually irregular in shape as they spread in a branch-like pathway through the less resistive parts of the bone (Figure 7). Over time, the cyst replaces bone tissue, destroys the cortex (Figure 6A), and gets to the surrounding soft tissues. So, the most common CT appearance is a single or multiple osteolytic lesion with serrated borders accompanied by cortical thinning [52]. Despite many other lytic lesions in the bone, most bone HDs do not show a periosteal reaction. A complicated lesion with superimposed bacterial infection may be a mimicker for neoplastic conditions. Lack of central enhancement could be helpful for differentiation [2, 56]. Intra-bone cysts rarely calcify (Figure 6) [57]. The involved bones in the T1 sequence have a low-to-moderate heterogeneous signal and high signal intensity in the T2 sequence.

Hydatid in the vertebrae can be similar to chronic spondylodiscitis of tuberculosis. Lack of osteoporosis and sclerosis in the involved bone, the absence of damage to the disk and vertebral bodies, paraspinal spread, subligamentous, subperiosteal, and adjacent rib involvement are the most common features of hydatid of the vertebrae [57, 58, 59].

The most widely used treatment for bone hydatidosis is a combination of surgery and systemic Albendazole [60]. Because of bone stiffness, HD in the bone cannot form typical spherical cysts which makes them more difficult to resect, with increased risk of recurrence after surgery [61]. Clavicle and rib HD better respond to surgical resection, but the spine, pelvis, and femoral HD are more prone to post-surgical relapse or sequels. Radiotherapy is another suggested alternative treatment, which can be considered in inoperable patients [62].

## 6 Intravascular

**Figure 8:**
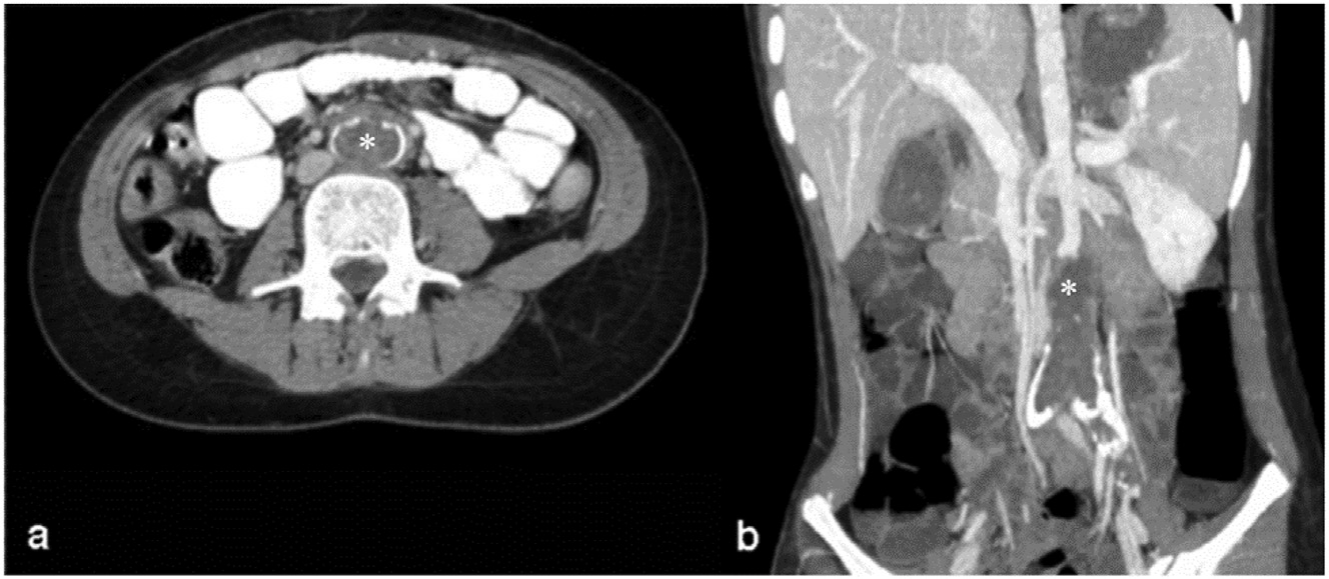
Axial (a) and coronal reformatted (b) contrast-enhanced abdominal CT scan in a 14-year-old girl shows an expansile filling defect with calcified walls in the abdominal aorta (Asterisk). The patient is a known case of generalized hydatidosis and this intra-aortic involvement has been stable for 2 years. (Photos are from our own patient(s) and they had not been published previously elsewhere.).

Arterial or venous involvement is extremely uncommon, even in endemic regions. There have been few reports of rupture of a liver hydatid cyst into the inferior vena cava [63, 64].There are also few reports of pseudoaneurysms caused by hydatid infection in the literature. The abdominal aorta is usually involved by one of the following mechanisms: a) direct invasion from retroperitoneal HD b) secondary to embolization from cardiac HD [65] (Figure 8).

## 7 Conclusion

Hydatid is a dynamic disease with many possible imaging appearances. This lesion can exist anywhere in the body with an available blood supply. HD less commonly involves the brain, orbit, muscle, bone, and vascular structure. Familiarity with typical clinical presentation, CT scan and MR imaging findings, and important mimickers facilitate the radiologic diagnosis and guiding appropriate treatment.

## Declarations

### Author contribution statement

All authors listed have significantly contributed to the development and the writing of this article.

### Funding statement

This research did not receive any specific grant from funding agencies in the public, commercial, or not-for-profit sectors.

### Data availability statement

Data will be made available on request.

### Declaration of interests statement

The authors declare no conflict of interest.

### Additional information

No additional information is available for this paper.
